# A 10-year-old male with chest wall Desmoid tumor – a rare tumor with unusual presentation

**DOI:** 10.1093/jscr/rjae007

**Published:** 2024-01-24

**Authors:** Arein A Abufara, Qusai A Alsalah, Anwar Yousef Jabari, Ahmad G Hammouri, Mohammad Najajreh

**Affiliations:** Faculty of Medicine, Palestine Polytechnic University, Hebron 9020000, Palestine; Faculty of Medicine, Palestine Polytechnic University, Hebron 9020000, Palestine; Faculty of Medicine, Palestine Polytechnic University, Hebron 9020000, Palestine; Radiology Department, Al-Ahli Hospital, Hebron 9020000, Palestine; Faculty of Medicine, Palestine Polytechnic University, Hebron 9020000, Palestine; Huda Al Masri Pediatric Cancer Department, Beit Jala Governmental Hospital, Bethlehem 9992100, Palestine

**Keywords:** desmoid tumor, chest wall, aggressive-fibromatoses, recurrence

## Abstract

Desmoid fibromatosis (DF) is a connective tissue tumor that grows aggressively in musculoaponeurotic tissues. It has an annual incidence rate of 2–4/million and is commonly seen in individuals aged 15 to 60 years, with female predominance. While it can occur in any body part, it is commonly found in the extremities, abdominal wall, and abdominal mesentery. But it rarely develops in the chest wall. The cause of the tumor is unknown. However, trauma to the tumor site has been identified in 25% of known cases. The primary treatment is surgical resection. Local recurrence after surgical excision is common. Our study highlights the case of a 12-year-old male patient diagnosed with a desmoid tumor on the chest wall 2 years ago, at the age of 10 years, after mild trauma. The tumor was successfully managed with surgery without radiotherapy treatment and no recurrence was observed in the last 2 years.

## Introduction

Desmoid fibromatosis (DF), also known as desmoid tumor, is a neoplasm that arises from fascia and musculoaponeurotic tissue with an annual incidence of 2–4/million accounts for roughly 3.5% of all fibrous tumors [1]. DF is most frequently diagnosed in individuals aged 15 to 60, exhibiting a higher incidence among females. They are less common in both pediatric and elderly populations [2]. DF can be classified based on location as extra-abdominal, abdominal, and intra-abdominal (60%, 25%, and 15%), respectively [1]. DF of the chest wall, which is extra-abdominal, accounts for only 10–20% of all DF cases [3]. The cause of the tumor is unknown. However, trauma to the tumor site has been identified in 25% of known cases [1]. Surgery is considered a potentially curative treatment [4], but local recurrence after surgical excision is expected [5]. Herein, we present a 12-year-old male patient who presented with a desmoid tumor on the chest wall 2 years ago – at age 10-year-old - after mild trauma managed successfully with surgery without recurrence in the last 2 years with close follow-up monthly with physical exam, chest X-ray and chest CT every 3–6 months. This report aims to shed light on this rare entity and alert physicians not to oversee these tumors in atypical cases.

## Case presentation

A 10-year-old male patient presented with a substantial mass located on the left side of the posterior chest wall. According to the child’s family, this mass appeared after mild trauma 2 years previously. They sought medical advice at the radiologist clinic where the chest ultrasound was done, and they were informed that the swelling would regress with time. But later, the family noticed that this mass was increasing in size gradually.

**Figure 1:**
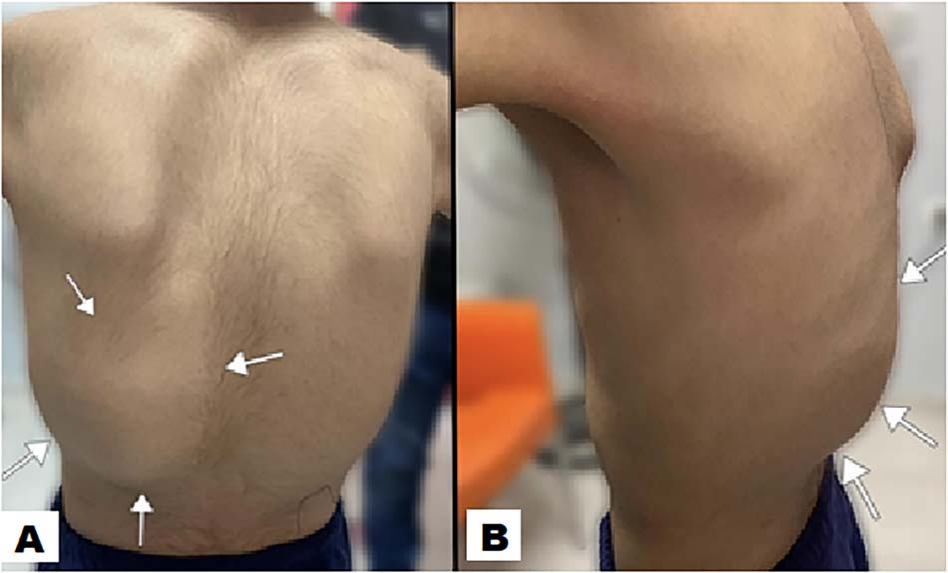
The posterior view illustrates a swelling (indicated by arrow) in the left lower posterior chest region, characterized by the absence of redness in the overlying skin (A). A lateral view offers a perspective of the swelling on the left posterior chest wall, measuring ~13 × 15 × 2 cm (B).

**Figure 2:**
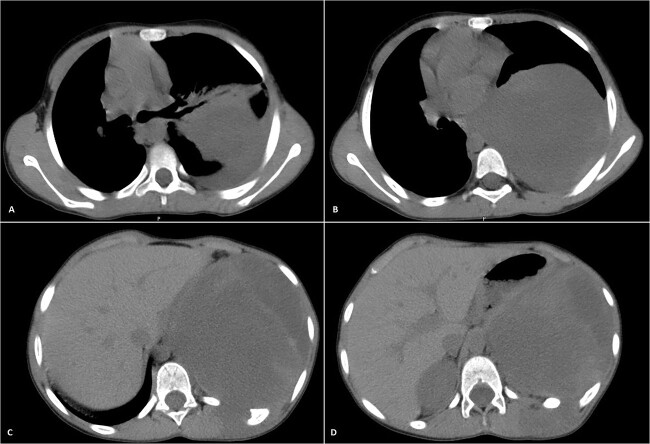
Selected axial CT images with mediastinal windowing showing multiple cuts of the patient’s chest. A huge relatively well-defined heterogeneous mass lesion is noted occupying the left lower hemi-thorax causing left lung volume loss and significant rightward mediastinal shift. No definite invasion of the adjacent ribs, however, remodeling of the lower aspect of the left posterior tenth rib is noted with the widening of the left tenth intercostal space and involvement of the left erector spinae muscles by the previously mentioned mass.

**Figure 3:**
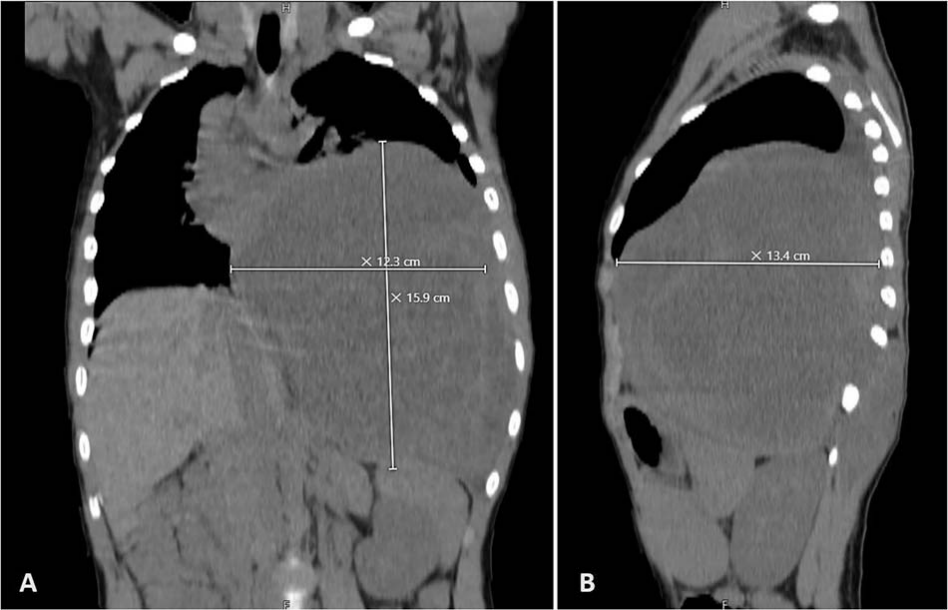
Selected coronal (A) and sagittal (B) cuts showing the size and the extension of the previously mentioned mass.

Physical examination unveiled a painless, solid mass characterized by hardness, emerging from the left lower chest and measuring 13 × 15 × 2 cm. This mass exhibited adherence to its surroundings, with no apparent pathological vasculature (Fig. 1). After this examination, a chest CT was conducted, revealing a huge, well-defined, and heterogeneous mass lesion occupying the left lower hemi-thorax. This imposing mass resulted in a noticeable reduction in left lung volume and a significant rightward mediastinal shift, measuring ~13.4 × 15.9 × 12.3 cm (Figs 2 and 3).

Following a CT-guided biopsy, immunohistochemical staining was performed, revealing positive results for β-catenin, while yielding negative results for S-100, Actin, desmin, and myogenin. These distinctive findings led to the histological diagnosis of a desmoid tumor. Subsequently, the decision was made to proceed with surgical intervention. The patient underwent a chest wall mass resection, followed by chest wall reconstruction utilizing cement and prolene mesh. Post-surgery, the pathology report confirmed a negative chest wall margin, indicating a successful excision of the tumor. Due to the significant correlation between familial adenomatous polyposis (FAP) and DF, the medical team pursued genetic consultation specifically to examine mutations in the APC gene. The outcome of the genetic study revealed a negative result, indicating the absence of mutations in the APC gene.

**Figure 4:**
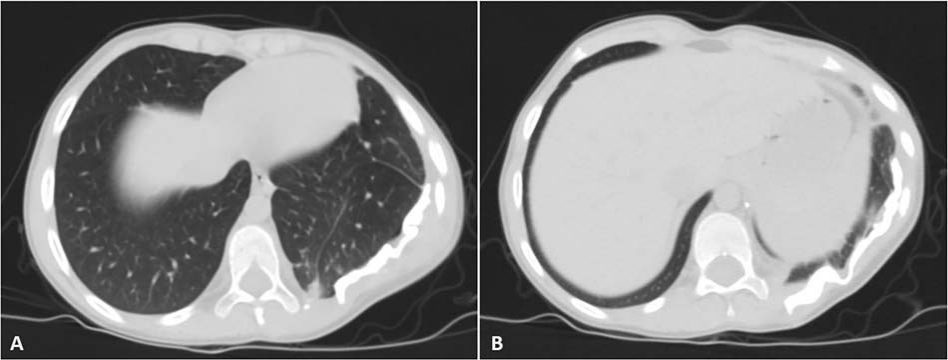
Selected axial cuts of the patient’s chest–lung window. The previously mentioned left posterior chest wall mass is no longer seen with post-operative changes noted.

**Figure 5:**
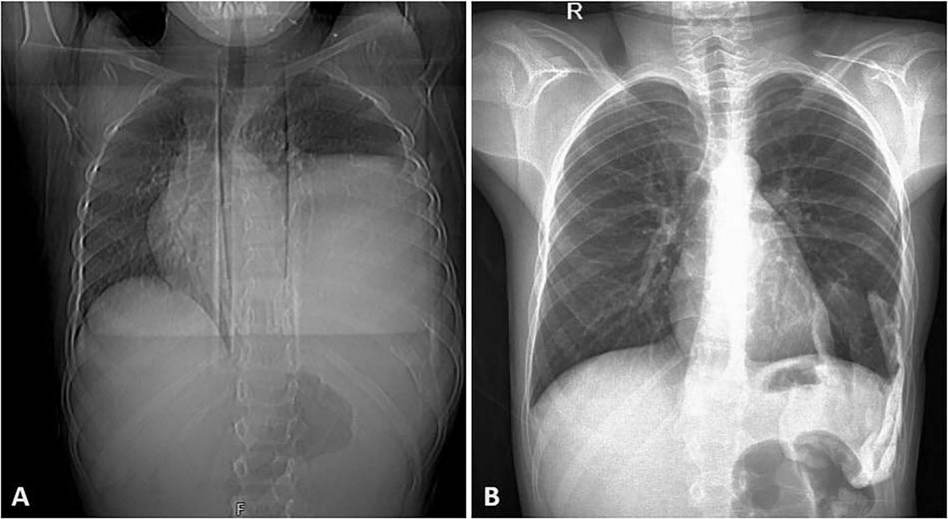
(A) Frontal chest radiograph of the same patient showing a left-sided opacity involving the left middle and lower lung zones and silhouetting the diaphragm and the left cardiac border in keeping with the known huge left posterior chest wall tumor. Widening of the posterior aspect of the left 10th intercostal space is noted. (B) Frontal chest radiograph taken during follow-up visits ~2 years following the surgical resection of the tumor, it shows re-expansion of the left lung with no residual or recurrent masses.

His postoperative course was uneventful. A chest CT was performed following the surgery, and the results revealed that the previous left lung mass had been completely removed without definite residue (Fig. 4). The patient is on close follow-up monthly with a physical exam, chest X-ray, and chest CT every 3–6 months. For the past two years, there have been no discernible clinical or radiological indicators of recurrence (Fig. 5).

## Discussion

Desmoid fibromatosis (DF) is a locally aggressive connective tissue tumor that appears in musculoaponeurotic tissues. It is also known as aggressive fibromatosis, deep fibromatosis, and desmoid tumor [1]. The most typical age range for the onset of DF is between 15 and 60, and females are more likely to experience it [2]. There is a dearth of information about desmoid tumors in children [6]. They account for 0.03% of all childhood neoplasms [7]. Although the cause is uncertain [6], Some well-known disorders, such as familial adenomatous polyposis (FAP), can put patients at risk for developing desmoid tumors. Additionally, localized trauma accounts for 25% of known cases in this site [1]. Also, prior surgery and excessive estrogen exposure may be linked to desmoids [8]. These tumors cannot spread by metastasis, they are characterized by slow, gradual growth, local invasion, and local recurrence after surgical excision [4]. Recurrence remains a problem following resection of desmoid tumors with as many as 50% of patients experiencing a recurrence within 5 years, factors associated with recurrence included age, tumor location, and margin status [8].

DF may affect any region but is most frequently found in the extremities, abdominal wall, and abdominal mesentery [9]. There haven’t been many specialized studies on tumors of the chest wall [4, 10]. It is a very rare site for these tumors [11]. The presence of a tumor was also found to be independently related to recurrence-free survival. Particularly, the risk of recurrence was increased for extra-abdominal tumors [8]. To diagnose, assess therapy effectiveness, and monitor these tumors, multimodal imaging techniques like ultrasonography, CT, and MR are helpful [9]. A particular diagnosis of benign chest wall lesions typically requires histological specimens, which can be successfully sampled under CT or ultrasound guidance [12].

Management of extra-abdominal desmoid tumors has been treated with surgery, radiation therapy, and chemotherapy [13]. While surgical resection remains central to the management of patients with desmoid tumors, the high rate of recurrence highlights the need for more effective adjuvant therapies [8], The reason for this is that complete removal of the tumor is uncommon [6].

## Conclusion

DF should be considered in the differential diagnosis of a posterior chest wall mass and a history of mild trauma in a male-child patient. The goal of its treatment is complete tumor excision and the avoidance of complications. Postoperative chemo-radiotherapy may be replaced with routine postoperative follow-up, and surgical removal of the mass is a wise option if it is possible to do so. This case report aims to shed light on this rare entity and alert physicians not to oversee these tumors in atypical cases.

## Data Availability

The data used to support the findings of this study are included in the article.
